# Supplementary material for: Janus particles with tunable patch symmetry and their assembly into chiral colloidal clusters
Source: Nat Commun. 2023 Dec 21;14:8494. doi: 10.1038/s41467-023-44154-6 (PMC10739893; doi:10.1038/s41467-023-44154-6)
Supplement: Supplementary file 1 — Supplementary information [file 41467_2023_44154_MOESM1_ESM.pdf]

Supplementary Information

Janus Particles with Tunable Patch Symmetry and their Assembly into Chiral Colloidal Clusters

Zhang *et al*.

Supplementary Figures

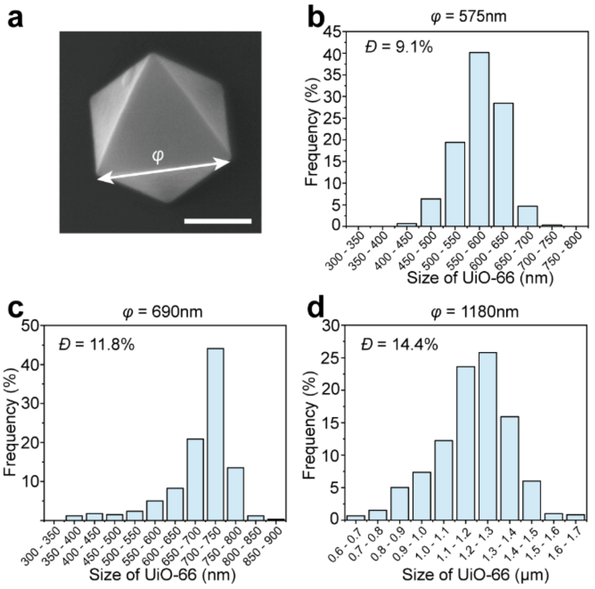

Supplementary Figure 1. Size dispersity of UiO-66 particles. a Scanning Electron Microscope (SEM) image of a UiO-66 particle showing its edge length *φ*. **b-d** The size distribution of UiO-66 particles. Scale bar: 500 nm.

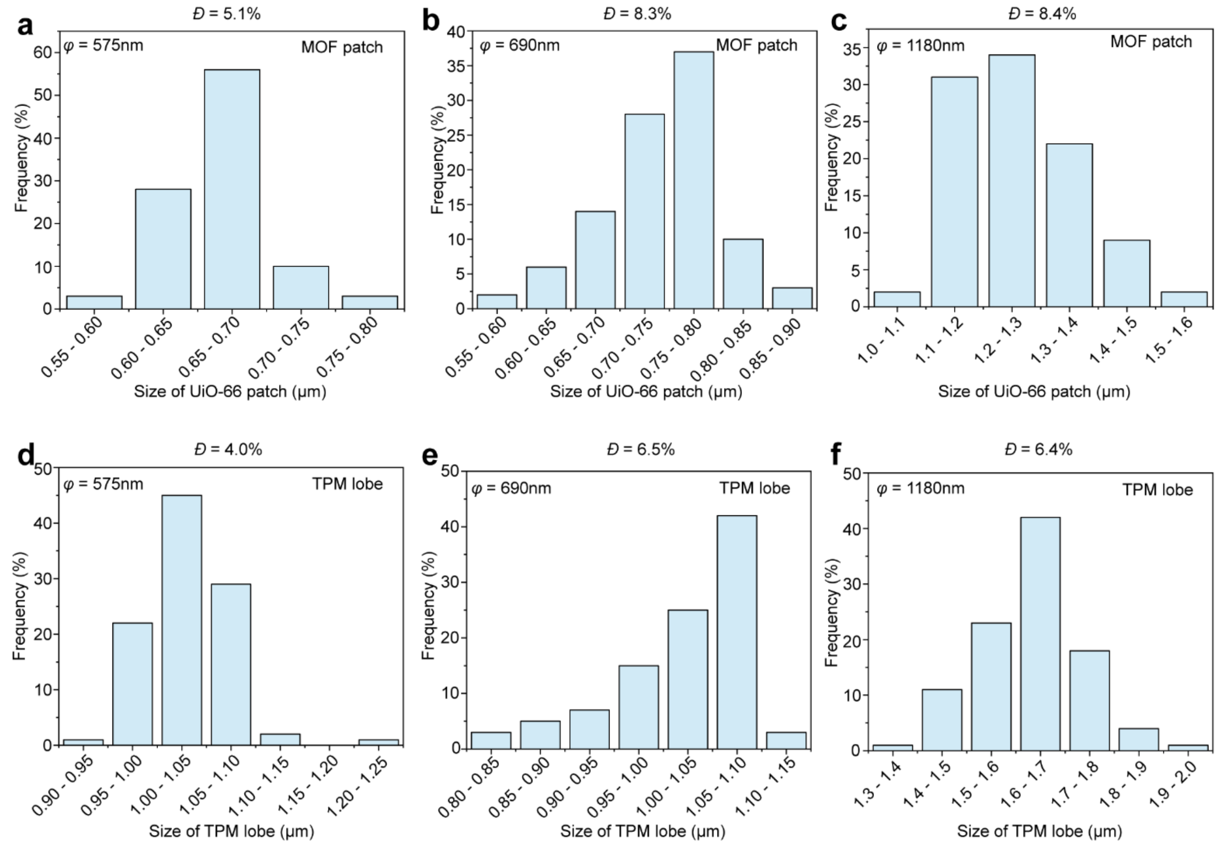

Supplementary Figure 2. Size dispersity of Janus particle S2. a-c Size distribution by measuring UiO-66 patches (*φ*). **d-f** Size distribution by measuring TPM lobes (*d*).

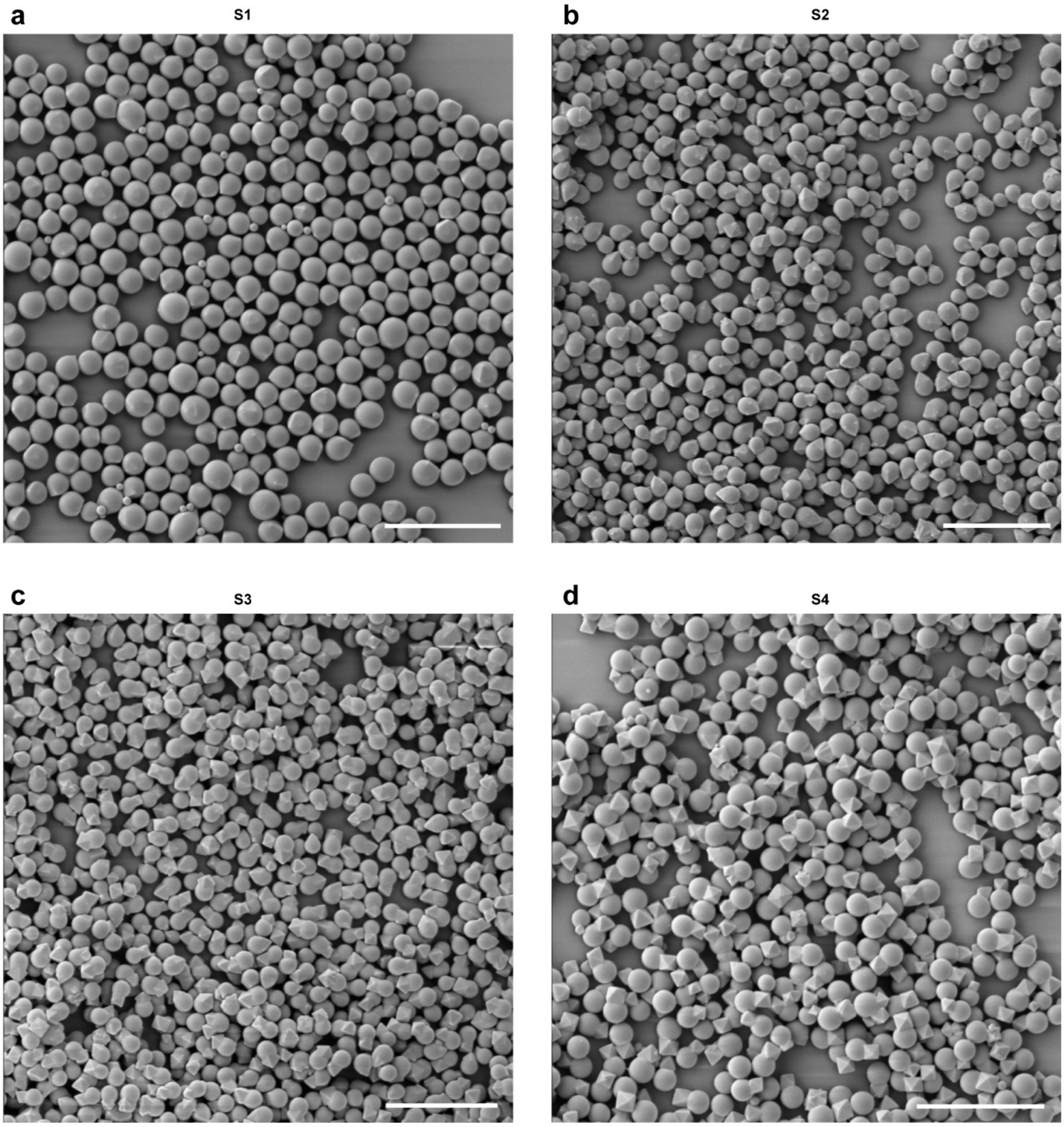

Supplementary Figure 3. Morphology of Janus particles on large scale. a-d SEM images of **S1** (**a**), **S2** (**b**), **S3** (**c**) and **S4** (**d**) in a large scale. Scale bars: 10 μm.

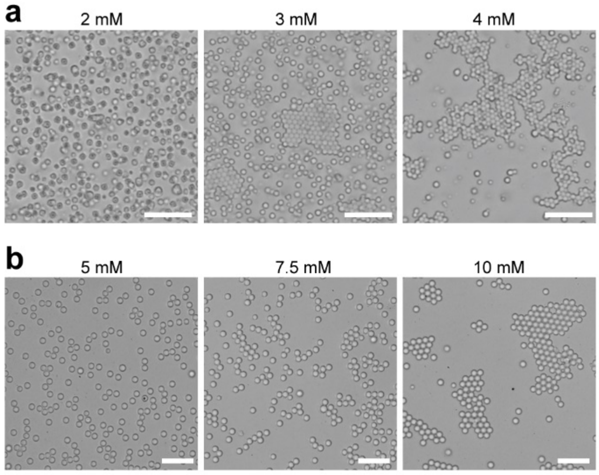

Supplementary Figure 4. Self-assembly in CTAC. **a-b** UiO-66 particles (**a**) and TPM particles (**b**) in different concentrations of CTAC. Scale bars: 10 μm.

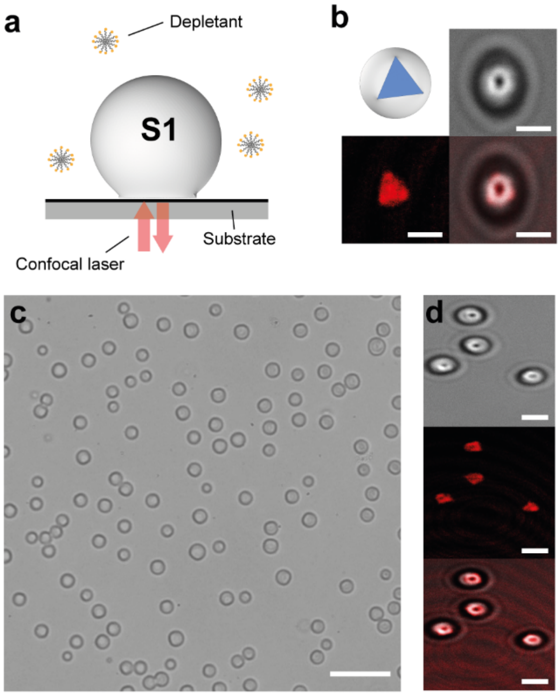

Supplementary Figure 5. S1 in 4 mM of CTAC. **a** Cartoon illustration of **S1** on substrate with depletion force. **b** Cartoon and confocal microscope images (transmission, reflected, and overlay) of **S1** on substrate. **c, d** Optical bright-field (**c**) and confocal images (**d**) of **S1** at a large view. Scale bar: 1 μm for (**b**), 10 μm for (**c**), 2 μm for (**d**).

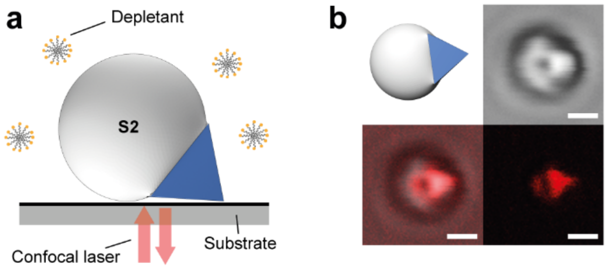

Supplementary Figure 6. S2 on substrate. **a** Cartoon illustration of **S2** on substrate with depletion force. The particle tends to stick to the substrate with one of its UiO-66 facets. **b** Cartoon and confocal microscope images (transmission, reflected, and overlay) of **S2**, indicating its orientation on the substrate. Scale bar: 1 μm for (**b**).

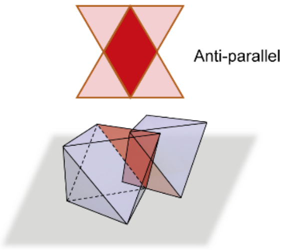

Supplementary Figure 7. Assembly of UiO-66 on substrate. The facets of UiO-66 contact each other in an antiparallel fashion.

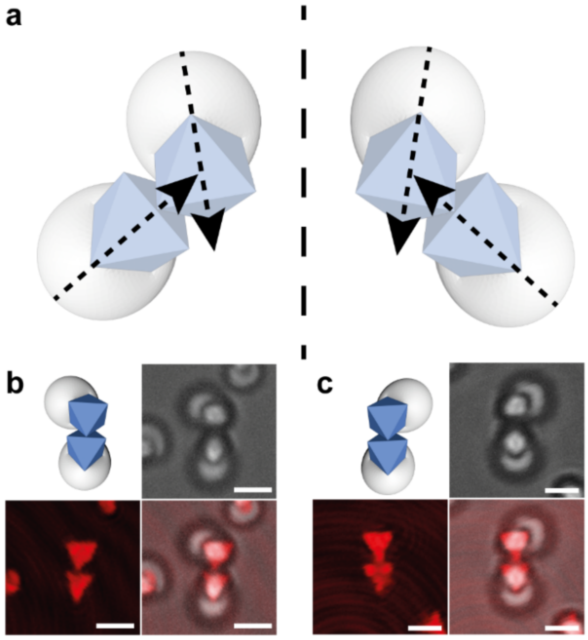

Supplementary Figure 8. **Chiral S3 dimers. a** The arrangement of C_2 axes in **S3** dimer. **b, c** Cartoon and confocal microscope images (transmission, reflected, and overlay) of chiral **S3** dimers. Scale bar: 1 μm for (**b, c**).

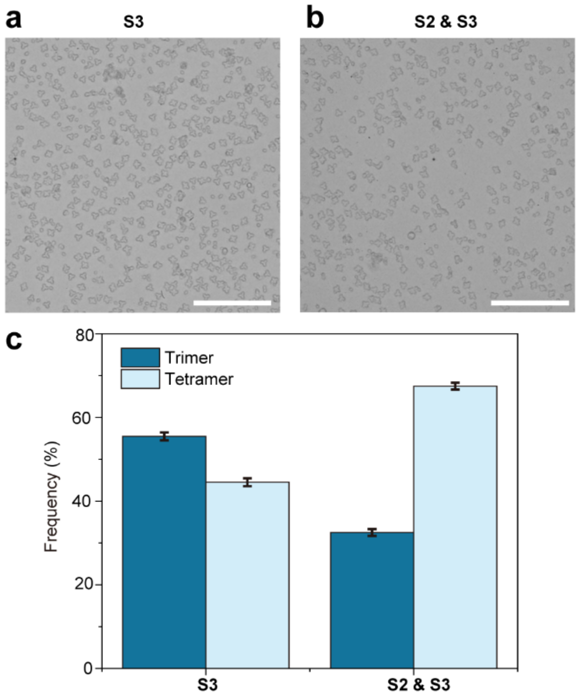

Supplementary Figure 9. Tetramer in S3 assembly. a, b Optical microscope images of assemblies of **S2** (**a**) and the mixture of **S2** and **S3** (**b**). **c** Frequency of trimers and tetramers in assemblies of **S3** and the mixture of **S2** and **S3**. Error bars are standard deviations. The measurement for frequency is repeated 3 times and around 1,000 particles are measured each time. Scale bars: 25 µm for (**a, b**).

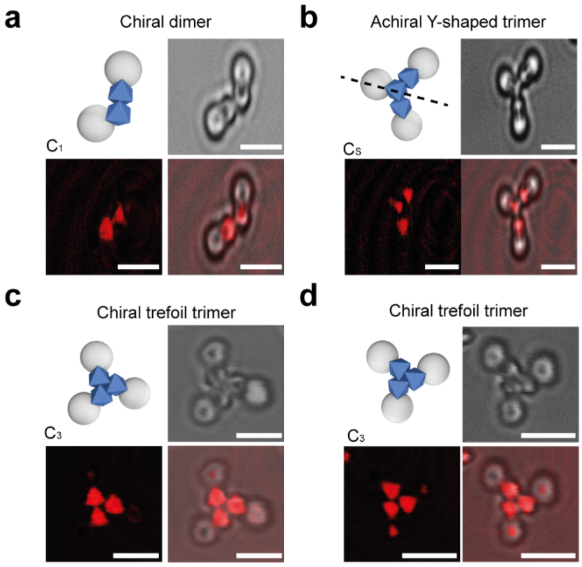

Supplementary Figure 10. Assemblies of S4. a-d Cartoon and confocal microscope images (transmission, reflected, and overlay) of chiral dimer (**a**), achiral Y-shaped trimer (**b**), chiral trefoil trimer (right-handed) (**c**), and chiral trefoil trimer (left-handed) (**d**). Scale bar: 3 μm for (**a-d**).

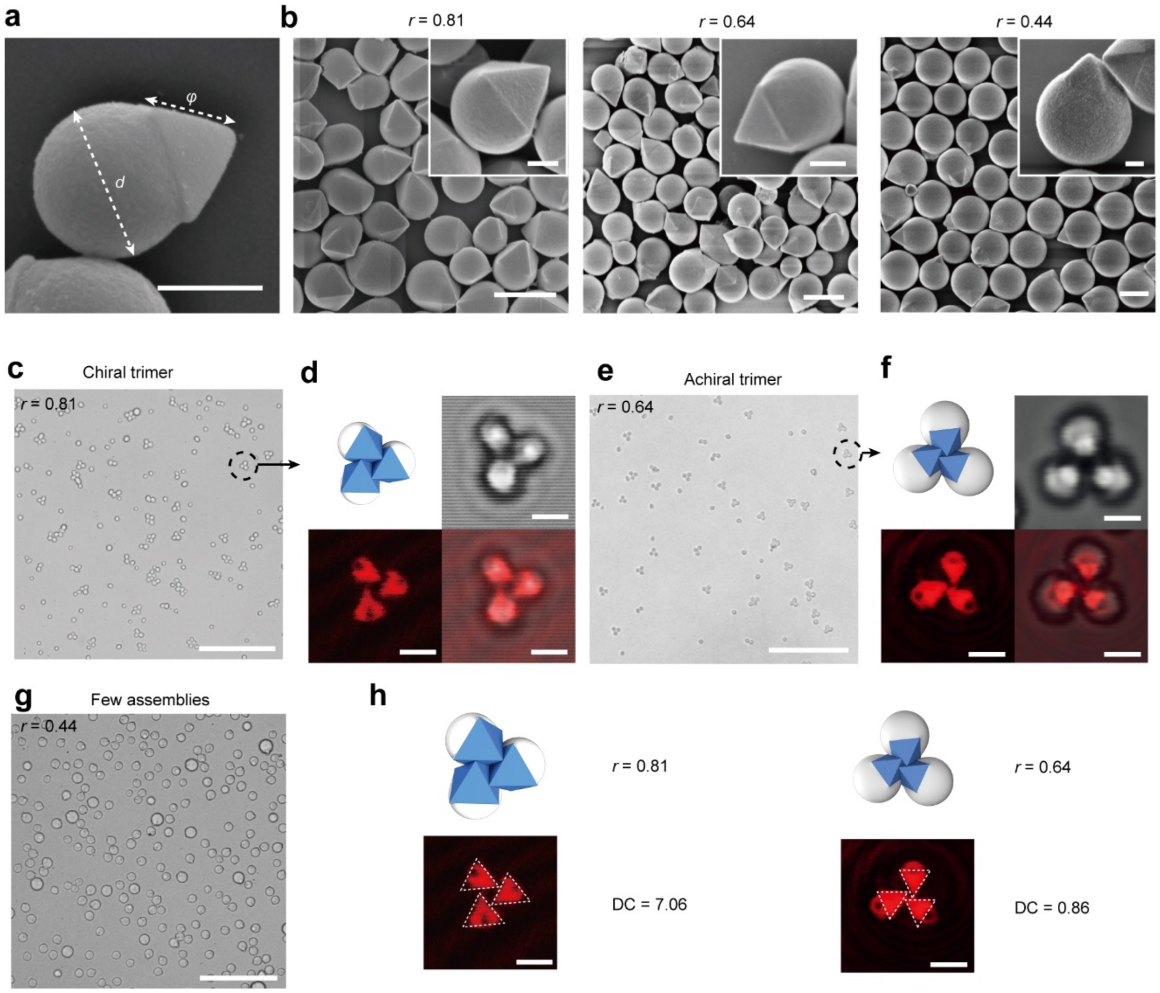

Supplementary Figure 11. The influence of patch ratio on the assembly of Janus particles (S2). a The illustration of patch ratio for Janus particles, where φ denotes the edge length of UiO-66 and d denotes the maximum diameter of the TPM lobe. **b** SEM images of **S2** with different patch ratios. **c, e, g** Optical microscope images showing the influence of the patch ratio on the assembly of **S2**. **d, f** Cartoon, optical microscope, confocal and overlay images of trimers found in (**c**) and (**e**), respectively. **h** Degree of chirality (DC) of trimers in (**d, f**). All the assemblies are conducted within the range of 3-6 mM CTAC. Scale bars: 1 μm for (**a**), 2 μm for large images and 500 nm for inset images in (**b**), 1.5 μm for (**d, f, h**), 25 μm for (**c, e, g**).

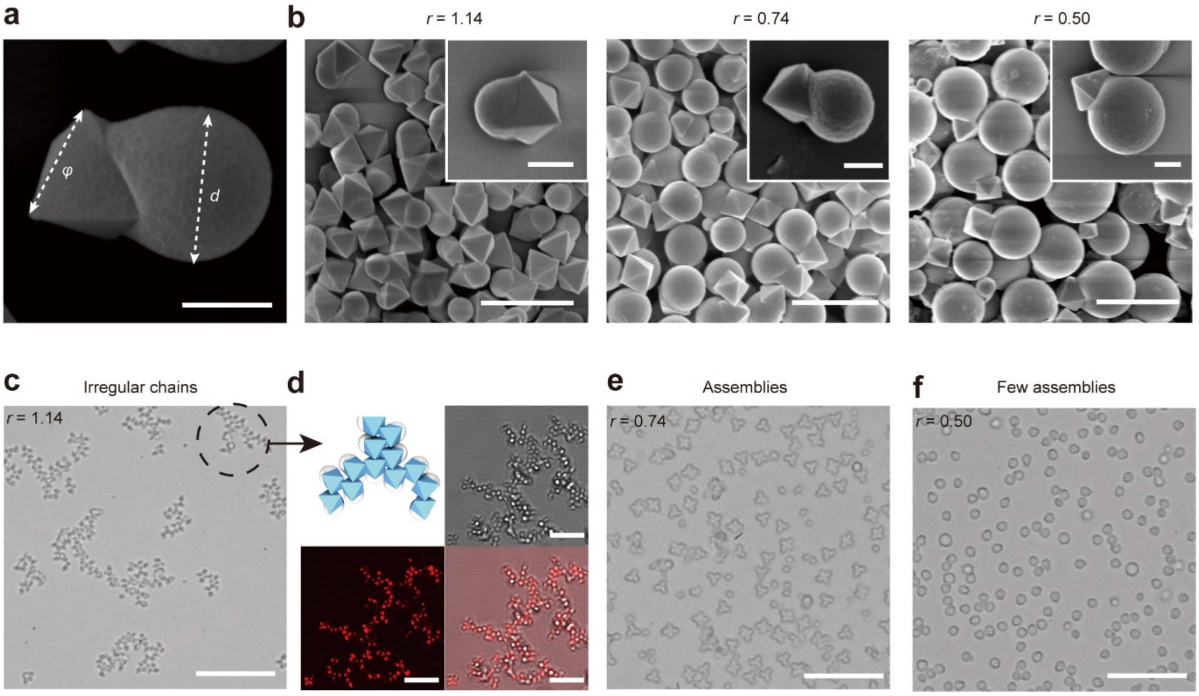

Supplementary Figure 12. The influence of patch ratio on the assembly of Janus particles (S3). a The illustration of patch ratio of **S3**, where *φ* denotes the edge length of UiO-66 and *d* denotes the maximum diameter of the TPM lobe. **b** SEM images of **S3** with different patch ratios. **c, e, f** Optical microscope images showing the influence of the patch ratio on the assembly. **d** Cartoon, optical microscope, confocal and overlay images of irregular chains found in (**c**). All the assemblies are conducted within the range of 3-6 mM CTAC. Scale bars: 1 μm for (**a**), 2 μm for large images and 500 nm for inset images in (**b**), 12.5 μm for (**c, e, f**), 5 μm for (**d**).

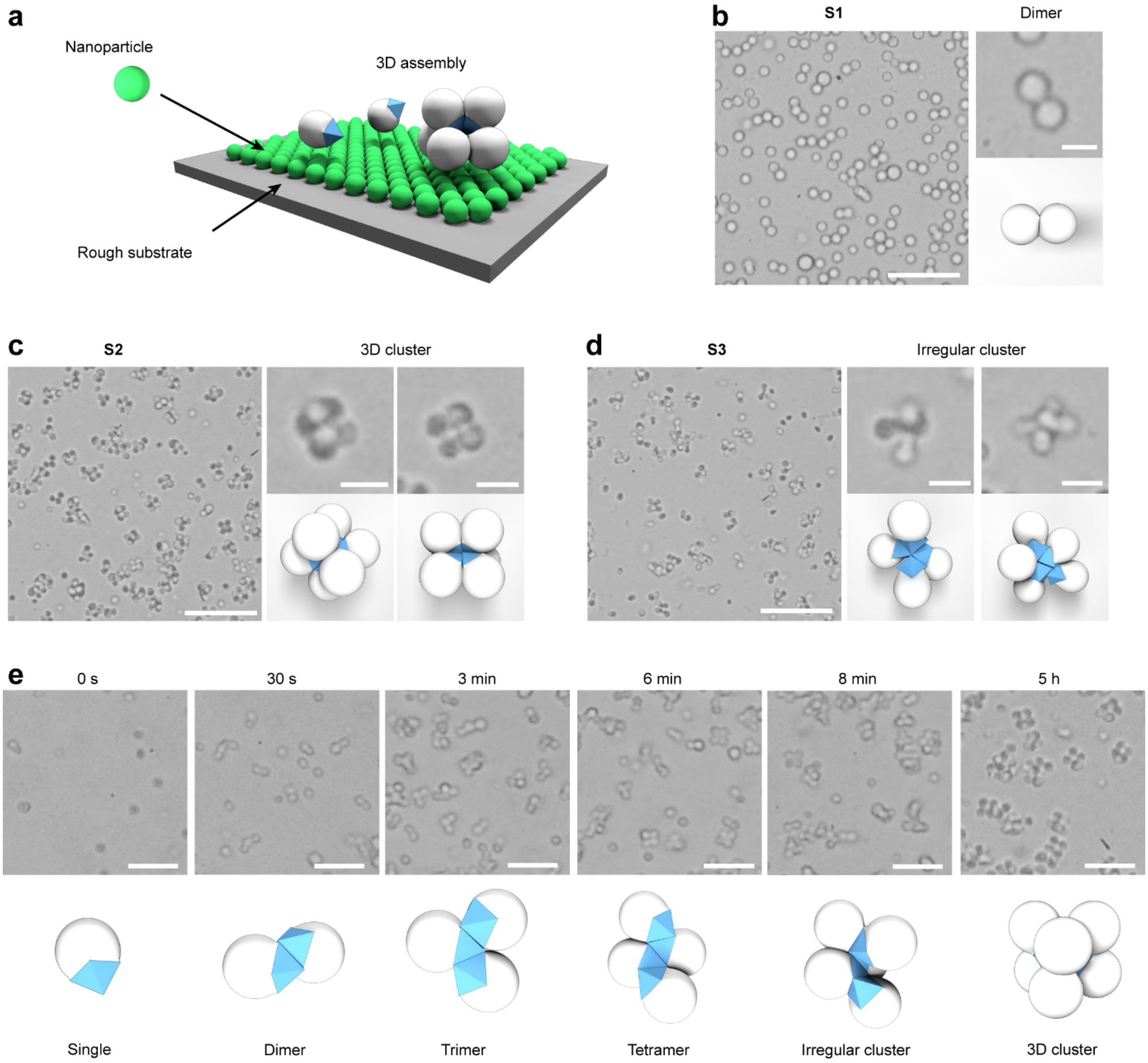

Supplementary Figure 13. Assemblies of Janus particles in 3D. a Illustration of the rough substrate and the 3D assembly of Janus particles. **b-d** Optical microscope and cartoon images of 3D assemblies of **S1** (**b**), **S2** (**c**) and **S3** (**d**). **e** Optic microscope and cartoon images showing the dynamics of the formation of 3D assemblies of **S2**. The lengthy formation may be ascribed to the many intermediate states as particles rearrange to explore the minimum energy state. Scale bar: 12.5 µm, and 2 µm for cropped images in (**b-d**), 6 µm for (**e**).

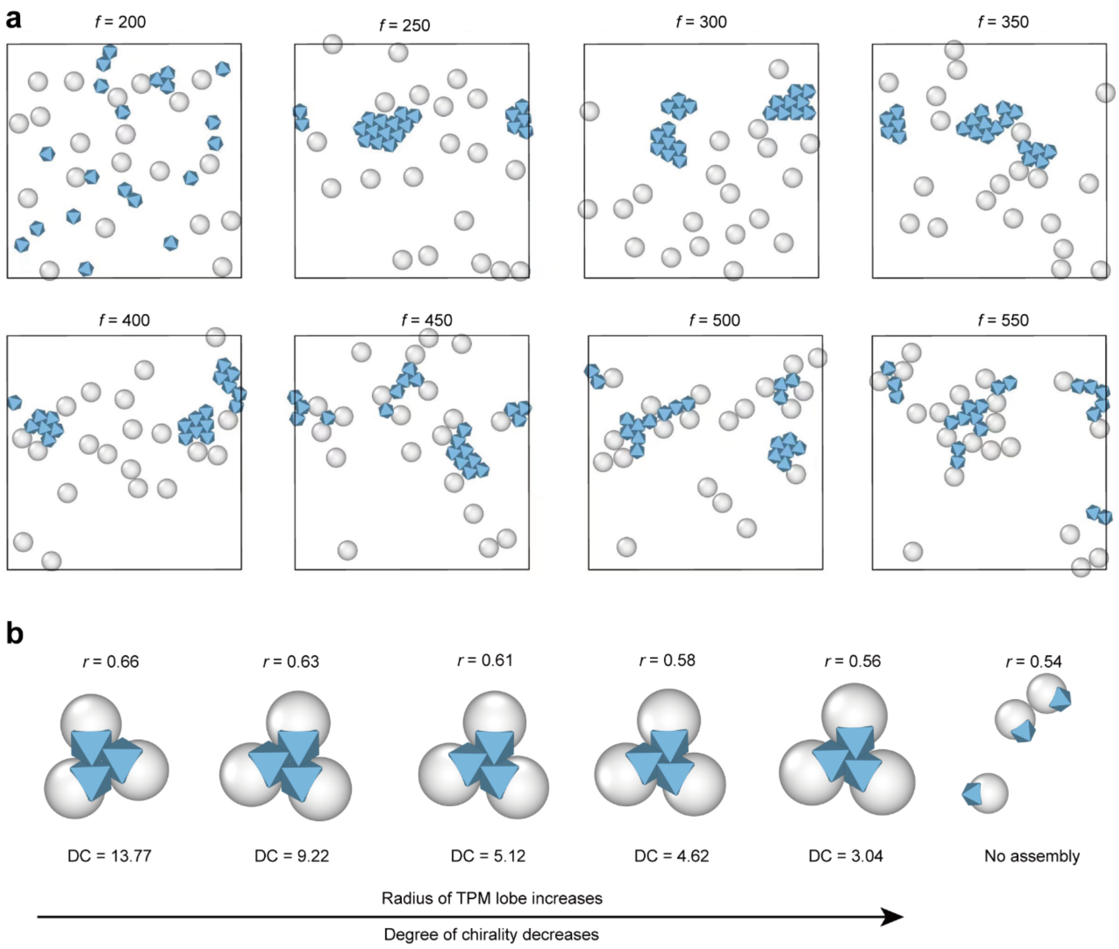

Supplementary Figure 14. a Selective assembly of UiO-66 particles and TPM particles in different settings of fugacity (*f*). **b** Self-assembly of **S2** with different patch ratios (*r*) when *f* = 350 arb. unit.

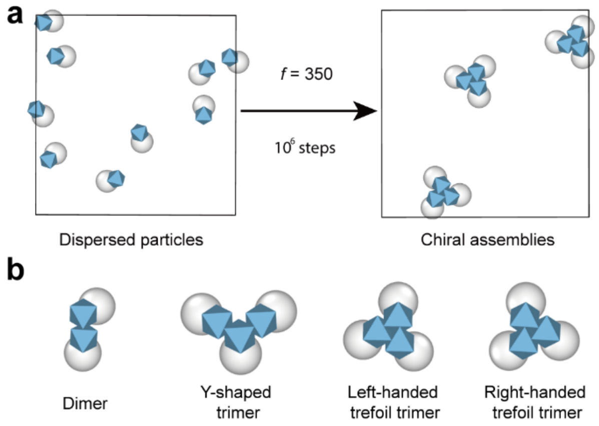

Supplementary Figure 15. Monte Carlo simulation of S3 assembly. a Self-assembly of **S3** when $f = 350$ arb. unit. **b** Clusters of **S3** by simulation.

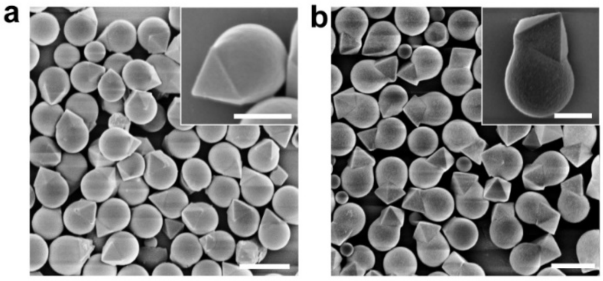

Supplementary Figure 16. SEM images of S2 (a) and S3 (b) for confocal characterization. Scale bar: 2 μm for large pictures, 500 nm for inset image in (**a**), and 1 μm for inset image in (**b**).

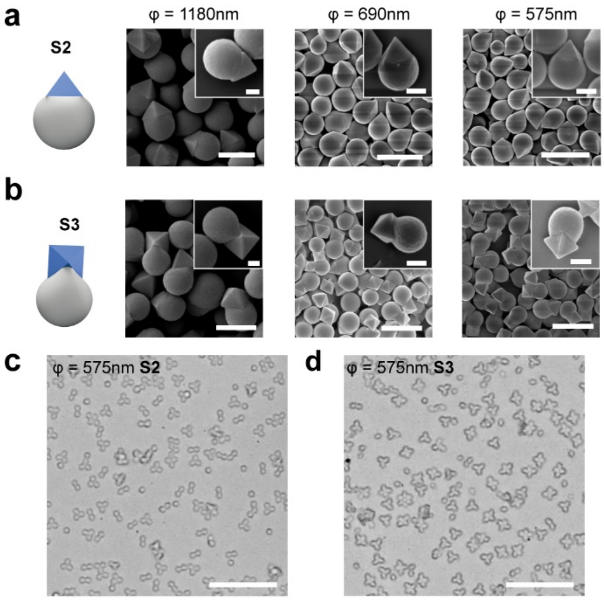

Supplementary Figure 17. Janus particles based on MOF (UiO-66) particles of different sizes. a, b Cartoon and SEM images of Janus particles **S2** (**a**) and **S3** (**b**) employing UiO-66 particles with different sizes. **c, d** Optical microscope images of assemblies of **S2** (**c**) and **S3** (**d**) synthesized from 575 nm UiO-66. Scale bars: 2 µm for large-scale images and 500 nm for inset images (**a, b**), 12.5 µm for (**c, d**).

Supplementary Notes

Supplementary Notes 1. Size dispersity of particles.

Generally, the UiO-66 MOF particles and the TPM particles have a narrow size distribution (or are considered monodisperse). Both particles have previously been utilized (by us and others) to assemble colloidal crystals[1-4]. In this paper, we have synthesized particles of three sizes, φ = 1180 nm, φ = 690 nm, and φ = 575 nm, (measuring the edge length φ of the octahedral particle) with size dispersity *Đ* = 14.4%, 11.8% and 9.1%, respectively (Supplementary Fig. 1).

After encapsulation by TPM oil (i.e., to make the Janus particle) and the subsequent wash steps, the *Đ* drops to *Đ* = 8.4%, 8.3%, and 5.1% (based on φ). Washing by repetitive centrifugation removed particles of small sizes, thereby improving the *Đ*. The TPM lobes of the Janus particles always have a low *Đ*. Taking **S2** for example, *Đ* (by diameter of TPM *d*) = 6.4%, 6.5%, and 4.0% for different MOF particles (Supplementary Fig. 2).

Because particles with *Đ* < 10% could be regarded as monodisperse[5]. Our particles are sufficiently uniform. Supplementary Fig. 3 shows the uniform particles from a large view.

Supplementary Notes 2. Tetramer in S3 assemblies.

As we show in Fig. 5, the major assembly of **S3** is trimer clusters, including mostly the chiral trimers and Y-shape trimer. With a wide open, the Y-shape trimer cannot adopt another particle to form the tetramer with a small quantity. Through further experimentation, we have found that the tetramer has to do with the small fraction of **S2** particles in the **S3** sample (Fig. 1h). When we purposely mix **S2** sample with **S3** sample, we can drastically increase the yield of tetramer (Supplementary Fig. 9), which supports our point. We note that in the synthesis, converting **S2** to **S3** is realized by addition of TX-100; insufficient amount of TX-100 would lead to a mixture of **S2** and **S3**.

Supplementary Notes 3. Absolute size of particles.

As mentioned above, apart from the φ = 1180 nm particles, which we originally used, we have explored the synthesis and assembly of Janus particles based on of UiO-66 particles of smaller sizes, φ = 690 and 575 nm. Their sizes are closer to the range (i.e., several 100s of nm) for possible optical/light applications in the future. In all cases, Janus particles can be synthesized, including the desired **S2** and **S3** configurations (shown in Supplementary Fig. 17). More importantly, they can assemble to form clusters (including chiral clusters) identical to that of larger Janus particles.

We also note a few points:

(1) With a smaller particle size, the particle overlap volume is reduced, so the concentration of depletants needed is higher for depletion-based assembly.
(2) For small particles, i.e., those based on φ = 575 UiO-66 particles, the purity of **S4** is compromised by possible detachment of the TPM droplet from the MOF particles.

(3) Because of TPM oil is formed through emulsion nucleation, their sizes have not been reported to be smaller than 100 nm (too small to be stable). The method is not yet suitable for making nanometer-sized Janus particles.

Supplementary References

1. Lyu D., Xu W., Payong J. E. L., Zhang T., Wang Y. Low-dimensional assemblies of metal-organic framework particles and mutually coordinated anisotropy. *Nat. Commun.* **13**, 3980 (2022).

2. Avci C.*, et al.* Self-assembly of polyhedral metal-organic framework particles into three-dimensional ordered superstructures. *Nat. Chem.* **10**, 78-84 (2017).

3. Wang Y.*, et al.* Synthetic Strategies Toward DNA-Coated Colloids that Crystallize. *J. Am. Chem. Soc.* **137**, 10760-10766 (2015).

4. Wang Y.*, et al.* Crystallization of DNA-coated colloids. *Nat. Commun.* **6**, 7253 (2015).

5. Clayton K. N., Salameh J. W., Wereley S. T., Kinzer-Ursem T. L. Physical characterization of nanoparticle size and surface modification using particle scattering diffusometry. *Biomicrofluidics* **10**, 054107 (2016).
