# Supplementary material for: Janus particles with tunable patch symmetry and their assembly into chiral colloidal clusters
Source: Nat Commun. 2023 Dec 21;14:8494. doi: 10.1038/s41467-023-44154-6 (PMC10739893; doi:10.1038/s41467-023-44154-6)
Supplement: Supplementary file 3 — Description of Additional Supplementary Files [file 41467_2023_44154_MOESM3_ESM.pdf]

Description of Additional Supplementary Files

File Name: Supplementary Movie 1

Description: **Transition from S2 to S3**. This movie shows the transition from **S2** to **S3** at increasing concentration of Triton X-100 (TX). TX at a high concentration is allowed to diffuse to the region of observation. The movie is played 10× of real time.

File Name: Supplementary Movie 2

Description: **Surface Evolver simulation of dewetting process on spherical particle**. This movie shows the dewetting process of liquid from the surface of a sphere by setting the contact angles from 10 to 160 degrees with a 5 degree interval.

File Name: Supplementary Movie 3

Description: **Surface Evolver simulation of transition from S1 to S2**. This movie shows the dewetting process of liquid from the surface of an octahedron by setting the contact angles from 50 to 60 degrees with a 5 degree interval.

File Name: Supplementary Movie 4

Description: **Assembly process of S3**. This movie shows the assembly process of **S3** particles on substrate in 4 mM CTAC. The movie is played 20× of real time.

File Name: Supplementary Movie 5

Description: **3D cluster of S2 on rough substrate**. This movie shows the 3D cluster of **S2** particles on a rough substrate after incubation in 5 mM CTAC for 5 hours.
